# Frequency Specific Optogenetic Stimulation of the Locus Coeruleus Induces Task-Relevant Plasticity in the Motor Cortex

**DOI:** 10.1523/JNEUROSCI.1528-23.2023

**Published:** 2024-02-14

**Authors:** Ching-Tzu Tseng, Hailey F. Welch, Ashley L. Gi, Erica Mina Kang, Tanushree Mamidi, Sahiti Pydimarri, Kritika Ramesh, Alfredo Sandoval, Jonathan E. Ploski, Catherine A. Thorn

**Affiliations:** ^1^Department of Neuroscience, The University of Texas at Dallas, Richardson 75080, Texas,; ^2^Department of Neurobiology, The University of Texas Medical Branch, Galveston 77555, Texas; ^3^Department of Neural and Behavioral Sciences, Penn State College of Medicine, Hershey 17033-0850, Pennsylvania

**Keywords:** locus ceruleus, motor learning, neocortex, neuroplasticity, noradrenaline, optogenetics

## Abstract

The locus ceruleus (LC) is the primary source of neocortical noradrenaline, which is known to be involved in diverse brain functions including sensory perception, attention, and learning. Previous studies have shown that LC stimulation paired with sensory experience can induce task-dependent plasticity in the sensory neocortex and in the hippocampus. However, it remains unknown whether LC activation similarly impacts neural representations in the agranular motor cortical regions that are responsible for movement planning and production. In this study, we test whether optogenetic stimulation of the LC paired with motor performance is sufficient to induce task-relevant plasticity in the somatotopic cortical motor map. Male and female TH-Cre + rats were trained on a skilled reaching lever-pressing task emphasizing the use of the proximal forelimb musculature, and a viral approach was used to selectively express ChR2 in noradrenergic LC neurons. Once animals reached criterial behavioral performance, they received five training sessions in which correct task performance was paired with optogenetic stimulation of the LC delivered at 3, 10, or 30 Hz. After the last stimulation session, motor cortical mapping was performed using intracortical microstimulation. Our results show that lever pressing paired with LC stimulation at 10 Hz, but not at 3 or 30 Hz, drove the expansion of the motor map representation of the task-relevant proximal FL musculature. These findings demonstrate that phasic, training-paired activation of the LC is sufficient to induce experience-dependent plasticity in the agranular motor cortex and that this LC-driven plasticity is highly dependent on the temporal dynamics of LC activation.

## Significance Statement

Noradrenergic input from the locus ceruleus (LC) is known to modulate cortical arousal, attention, and sensory perception. The impacts of noradrenergic signaling on motor cortical networks, however, remain relatively poorly understood. In the current study, we demonstrate that brief, movement-paired LC activation is sufficient to induce experience-dependent plasticity in the motor cortex. Further, this LC-driven motor cortical plasticity is highly dependent on the frequency of LC stimulation, exhibiting an inverted U-shaped relationship with increasing stimulation frequency. These findings point to the temporal dynamics of noradrenergic signaling as an important driver of motor cortical network optimization and experience-dependent plasticity, with implications for targeting this key neuromodulatory system to aid patients with motor deficits.

## Introduction

The neocortex receives topographically organized noradrenergic input from the locus ceruleus (LC; Levitt and Moore, 1978; Loughlin et al., 1982; Agster et al., 2013; Chandler, 2016). Noradrenaline (NA) has long been known to modulate neocortical activity (Krnjevic and Phillis, 1963; Foote et al., 1975; Salgado et al., 2016; Vitrac and Benoit-Marandmarianne, 2017) and to play a key role in regulating brain arousal states (Berridge, 2008; Carter et al., 2010; Sara and Bouret, 2012), modulating sensory processing and perception (Jacob and Nienborg, 2018; McBurney-Lin et al., 2019), and mediating attention, behavioral flexibility, and working memory (Aston-Jones and Cohen, 2005; Arnsten, 2011; Thiele and Bellgrove, 2018). In rat auditory cortex, for example, repeated pairing of LC stimulation with a specific tone frequency shifts neural tuning curves and improves perceptual learning (Manunta and Edeline, 2004; Edeline et al., 2011; Martins and Froemke, 2015; Glennon et al., 2019). LC stimulation similarly enhances thalamocortical representations of sensory information and improves animals’ performance on tasks requiring visual or somatosensory discrimination (Devilbiss and Waterhouse, 2011; Mizuyama et al., 2016; Gelbard-Sagiv et al., 2018; Rodenkirch et al., 2019; Xiang et al., 2019; McBurney-Lin et al., 2022) or spatial learning (Wagatsuma et al., 2017; Grella et al., 2019; Kaufman et al., 2020).

Other studies demonstrate that increases in noradrenergic signaling result in enhanced prefrontal cortical excitability (Barth et al., 2007, 2008; Carr et al., 2007; Wang et al., 2007; Zhang et al., 2013), with profound impacts on attention and working memory performance (Aston-Jones and Cohen, 2005; Kane et al., 2017; Cope et al., 2019; McBurney-Lin et al., 2022; Cerpa et al., 2023). Moreover, these attention-enhancing effects are typically found to depend on the precise temporal dynamics of noradrenergic signaling (Usher et al., 1999; Berridge and Waterhouse, 2003; Totah et al., 2019; Noei et al., 2022; Xiang et al., 2023). In the classic model, low-frequency tonic firing of the LC (ca. 1−5 Hz) is associated with wakefulness and behavioral flexibility and with poor behavioral performance on tasks requiring focused attention (Usher et al., 1999; Aston-Jones and Cohen, 2005; Kane et al., 2017). By contrast, task-relevant bursts of higher-frequency LC firing (ca. 8–15 Hz) are seen during periods of optimal behavioral performance (Usher et al., 1999; Aston-Jones and Cohen, 2005; Howells et al., 2012). Sustained LC firing at these higher frequencies, however, is associated with a decline in focused attention, suggesting an inverted U-shaped relationship exists between noradrenergic activity and cortex-dependent behavioral performance (Aston-Jones and Cohen, 2005; Levy, 2009; Devilbiss, 2019).

In comparison to other neocortical regions, the impact of noradrenergic signaling in M1 remains underexplored (Waterhouse et al., 2022). Prior studies have demonstrated that NA modulates M1 circuit dynamics (Matsumura et al., 1990; Goldstein, 2006; Meintzschel and Ziemann, 2006; Sheets et al., 2011; Schiemann et al., 2015; Vitrac and Benoit-Marandmarianne, 2017) and that noradrenergic signaling modulates motor performance (Schiemann et al., 2015; Breton-Provencher et al., 2022) and contributes to motor cortical map plasticity (Hulsey et al., 2019; Tseng et al., 2021). It is not known, however, whether pairing of phasic LC activity with motor performance can induce cortical map plasticity in agranular M1, similar to that which is seen in the granular sensory cortices. Nor is it known whether LC-driven cortical motor map plasticity exhibits an inverted U-shaped dose dependence, as is prominently observed in prefrontal cortical areas. To address these questions, we used an optogenetic approach to stimulate the LC at 3, 10, or 30 Hz while rats performed a well-learned skilled reaching lever-press task. We found that motor training paired with brief LC stimulation at 10 Hz, but not at lower (3 Hz) or higher (30 Hz) stimulation frequencies, generated task-relevant motor cortical map plasticity. These results suggest that phasic activation of the LC is indeed sufficient to induce experience-dependent map plasticity in agranular M1 and that this form of LC-driven cortical plasticity is highly dependent on the precise temporal dynamics of LC signaling.

## Materials and Methods

All procedures were conducted in accordance with the National Institutes of Health Guide for the Care and Use of Laboratory Animals and were approved by the University of Texas Institutional Animal Care and Use Committee.

### Experimental design

**Figure 1.**
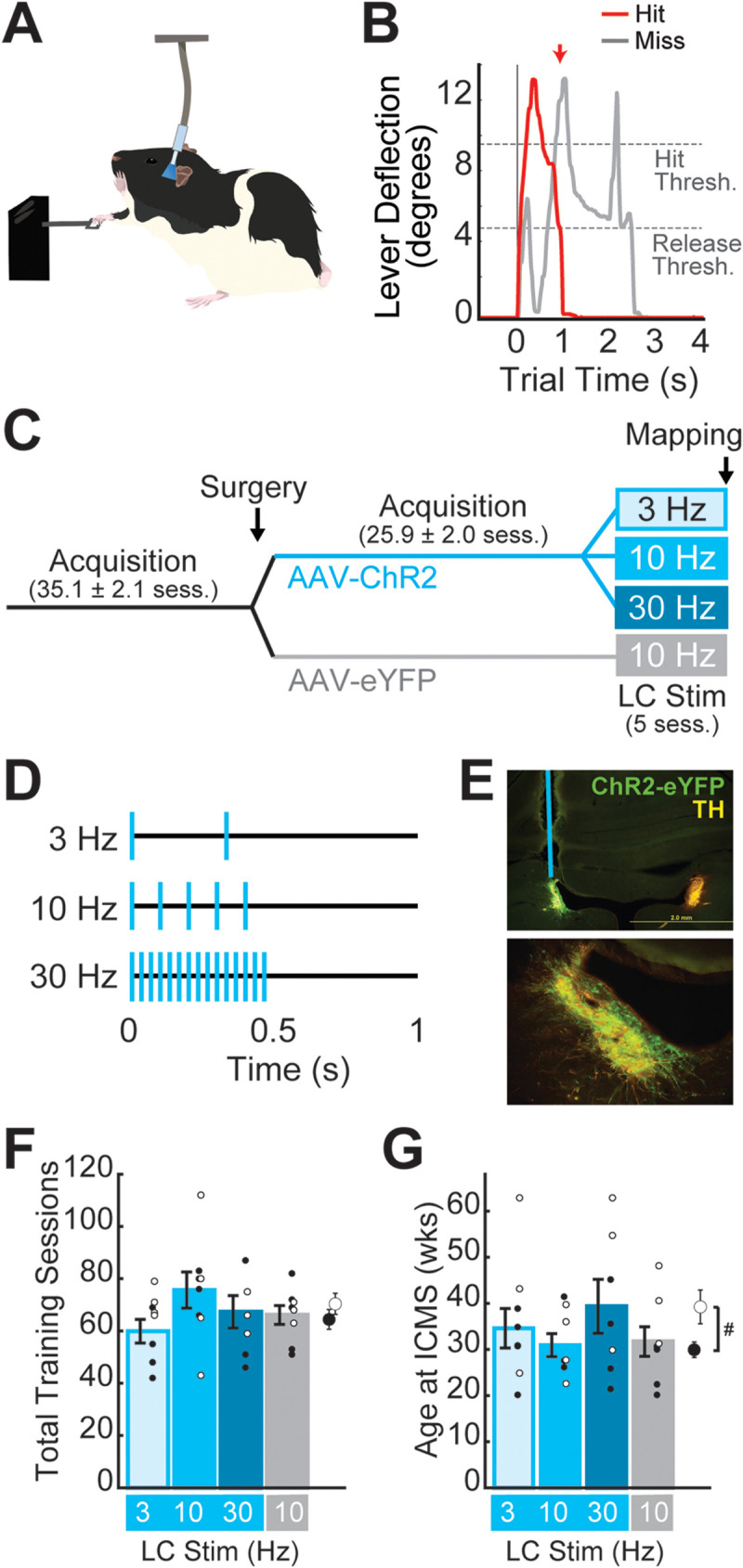
Experimental design. ***A***, Cartoon illustrating the skilled reaching lever-press task paired with optogenetic stimulation of the LC. ***B***, Example rewarded (hit, red) and unrewarded (miss, gray) trial from a single session. Rats were required to press the lever past the “Hit Threshold” (9.5° below horizontal) and return it to less than 4.75° from horizontal (“Release Threshold”) within a 2 s time window to receive a food reward. Unrewarded miss trials were those in which rats either failed to reach the hit threshold or failed to release the lever within the required time window. ***C***, Experimental timeline. Male and female Long–Evans TH-Cre + rats began training on the lever-press task prior to the surgical infusion of eYFP-tagged Cre-dependent virus and the implantation of an optical fiber over the left LC. After surgery, rats were trained until behavioral criteria were reached and then dynamically allocated to a treatment group. During LC Stim treatment, rats received five training sessions in which 3, 10, or 30 Hz LC stimulation was paired with each correct lever press. Cortical motor mapping was performed within 24 h of the last training-paired stimulation session. ***D***, Optogenetic stimulation consisted of a 0.5 s train of 470 nm laser pulses delivered at 3, 10, or 30 Hz. ***E***, 4× (top) and 20× (bottom) images from one subject showing virus expression and the placement of the optical fiber in the left LC. Slices were stained for GFP (ChR2-eYFP, green) and tyrosine hydroxylase (TH, yellow). ***F***,***G***, Total training sessions performed (***F***) and age at ICMS mapping (***G***) were balanced across treatment groups.

To test whether noradrenergic LC stimulation is sufficient to induce task-relevant cortical reorganization, we adapted a training-paired stimulation protocol previously found to induce M1 map plasticity when vagus nerve stimulation (VNS) was applied (Tseng et al., 2020, 2021). TH-Cre rats were first trained to perform a skilled reaching lever-press task with their right forelimb (FL; Fig. 1*A*,*B*). Once animals learned the association of lever press with food rewards, an eYFP-tagged, Cre-dependent channelrhodopsin (ChR2) virus (AAV8-EF1a-DIO-hChR2(H134R)-EYFP) or control virus (AAV8-EF1a-DIO-EYFP) was infused (vol. = 1 μl, titer = ∼1 × 10^13^ GC/ml) into subjects’ left LC (AP, −10 mm; ML, −1.25 mm; DV, −7.0 mm from Bregma) and an optical fiber was implanted just above the injection site. After recovering from surgery, rats returned to training until the criterial behavioral performance was established. Rats that received ChR2 virus infusions were then dynamically allocated (Pocock and Simon, 1975) to one of three stimulation groups: 3 Hz (*n* = 6 male, 6 female); 10 Hz (*n* = 6 male, 5 female); and 30 Hz (*n* = 6 male, 4 female). Stimulation frequencies were chosen to span a broad range of LC firing modes, including typical physiological tonic (3 Hz) and phasic (10 Hz) firing of LC neurons, as well as a super-physiological or “overstimulated” firing mode (30 Hz) hypothesized to be relevant to stimulation-based stroke therapies such as VNS (Hulsey et al., 2017; Hays et al., 2023). Rats in the control virus group (*n* = 7 male, 4 female) received 10 Hz stimulation. The experimental timeline and treatment groups are illustrated in Figure 1*C*. During the final five sessions of training, each correct lever press was paired with LC stimulation, which consisted of a 0.5 s train of 3, 10, or 30 Hz laser (473 nm) pulses (Fig. 1*D*). Within 24 h of the last training-paired LC stimulation session, intracortical microstimulation (ICMS) was performed to acquire the somatotopic cortical motor map in left M1. Finally, immunohistochemistry was used to confirm the expression of the eYFP-tagged viruses and the placement of the optical fibers (Fig. 1*E*). Of the 44 rats that completed training and ICMS, 3 were excluded from further data analysis due to incorrect fiber placements, and 9 were excluded due to inadequate virus expression. A total of 32 rats were included in the behavioral study, in the following treatment groups: 3 Hz stimulation (5 male + 4 female), 10 Hz stimulation (4 male + 4 female), 30 Hz stimulation (4 male + 3 female), and eYFP control (5 male + 3 female). Seven additional training-naive adult rats (4 male + 3 female) underwent ICMS mapping to control for the effects of lever training on M1 map structures.

To validate the frequency-dependent optogenetic activation of noradrenergic neurons in the LC, in vivo electrophysiology recordings were performed under anesthesia in three additional rats. Single-unit recording and data analysis procedures are detailed below (see *Electrophysiology*).

### Animal subjects

A total of 25 male and 19 female TH-Cre Long–Evans rats (RRID RRRC_00659; Witten et al., 2011), 9–45 weeks old at the study start, were included in the lever-press experiments. Adult rats were paired housed before surgery and single housed after surgery in a 12:12 h reversed light cycle room (lights on at 6:00 pm). All experimental procedures were performed during the animals’ dark cycle. Rats that underwent behavioral training were food restricted to not less than 90% of their free-feeding weights. A training-naive group consisting of four male (2 TH-Cre + 2 wild-type littermates) and three TH-Cre female rats, aged 24–29 weeks old, also underwent ICMS motor mapping. In vivo electrophysiology recordings were performed in three female TH-Cre rats, aged 33–68 weeks old at recording. All rats were bred in-house and genotyped at weaning according to vendor protocols.

### Behavioral training

Prior to the start of behavioral training, rats were acclimated to handling during 10 min acclimation sessions for 5–7 d. Rats were then trained to perform a previously published automated skilled reaching lever-press task (Tseng et al., 2020, 2021; Brougher et al., 2021). The task required subjects to fully depress (to >9.5° below horizontal) and release (to <4.75° below horizontal) a lever positioned 2 cm outside a MotoTrak behavioral training booth (Vulintus) within a 2 s time window to receive a 45 mg food pellet reward (Bio-Serv #F0021). The MotoTrak booth consisted of an acrylic cage (30 cm × 13 cm × 25 cm) with a window through which the rats accessed the lever. A cage divider was positioned next to the window to ensure that the rats performed the lever-press task with their right FL.

Rats underwent daily 1 h behavioral training sessions, 5 d per week. The training was conducted in stages. During 2 initial habituation sessions, the lever was positioned 1 cm inside the training booth, and lever presses were rewarded using an adaptive threshold. The initial press threshold was set to 1° from horizontal, and the threshold was incremented (or decremented) by 0.5° if the median lever press over the last 20 trials exceeded (or failed to exceed) the current threshold. After habituation, in stage 1, the lever was located 1 cm inside the booth and rats were rewarded for making progressively larger lever depressions. In stage 1, pressing thresholds were incremented by 0.5° if the median press over the last 20 trials exceeded the current threshold (as during habituation), but thresholds were no longer decremented. Once rats were able to fully depress the lever for 100 trials/day for 2 consecutive days, the rats progressed to stage 2. In stage 2, the lever was progressively moved from the interior to the exterior of the booth, in 0.5 cm increments every 30 successful trials, until the final position of 2.0 cm outside the booth was reached. During stage 2, rats were rewarded upon release of the lever following a full depression. Rats typically received virus injection and optical fiber implantation surgery early in stage 2, after they performed more than 100 trials/day for at least 2 consecutive days. After surgical recovery, daily training continued until pre-stimulation performance criteria were reached: at least 100 trials and at least 60% correct per day for at least 4 of 5 consecutive training days. During late stage 2 acquisition sessions, after the final −2.0 cm lever position was reached, rats were connected to the fiber-optic patch cable during training to further acclimate to the stimulation environment (no stimulation was delivered).

### Optogenetic stimulation

Once the criterial behavioral performance was reached, rats with ChR2 virus infusions were dynamically assigned to receive either 3, 10, or 30 Hz stimulation; rats with eYFP virus infusions received 10 Hz stimulation as a negative control. All subjects underwent five final training-paired LC stimulation sessions (1/day) in which each correct lever press was paired with a 0.5 s train of 3, 10, or 30 Hz laser stimulation (Shanghai Laser and Optics; *λ* = 473 nm; pulse width = 10 ms; power at fiber tips = 10–16 mW).

### Surgery

Rats were anesthetized with ketamine hydrochloride (70 mg/kg) and xylazine (5 mg/kg) injected intraperitoneally. To reduce pain and inflammation, rats were injected with carprofen (Zoetis) subcutaneously prior to all surgical procedures. Rats were placed in a stereotaxic frame, and an incision was made to expose bregma and lambda. Six anchor screws (Stoelting) were inserted into the skull anterior to the targeted injection site. A small craniotomy was made to target the LC (AP, −12 mm; ML, −1.25 mm from bregma), and a 32-gauge infusion needle attached to a 10 μl Hamilton syringe (Hamilton) was stereotaxically lowered to the target depth (5.5 mm below the pial surface) at an angle of 20° posterior to vertical (Quinlan et al., 2019). A total volume of 1 μl of virus was infused at a rate of 0.1 μl/min. The needle was held in place for 5 min after the completion of the infusion to allow the virus to diffuse, then slowly raised and removed from the brain. A Ø400 µm core, 0.39 NA, fiber-optic cannula (Thorlabs) was then stereotaxically placed just above the injection site (5.4–5.45 mm below the pial surface) and cemented in place with acrylic. The incision was closed with sutures, and a topical antibiotic cream was applied to the incision site. Rats received 3–7 d of recovery before training was resumed.

### Viruses

Adeno-associated viruses (AAVs) were used to deliver and express channelrhodopsin and eYFP in a Cre-dependent manner. Specifically, plasmids encoding pAAV-EF1a-double floxed-hChR2(H134R)-EYFP-WPRE-HGHpA (RRID Addgene_20298) and pAAV-Ef1a-DIO-EYFP (RRID Addgene_27056) were gifts from Karl Deisseroth obtained from Addgene. These plasmids were used to create AAVs pseudotyped as AAV8 using a triple transfection, helper-free method as previously described (Holehonnur et al., 2014). Plasmids encoding pRC-AAV8 (Penn Vector Core, University of Pennsylvania), pHelper, and AAV2 genome plasmids were transfected into 293FT cells (Invitrogen, catalog #R70007) using Turbofect (Thermo Scientific) following the manufacturer's instructions. Seventy-two hours later, the cells were harvested, and AAVs were purified on an iodixanol step gradient via ultracentrifugation and underwent buffer exchange (1× PBS, 0.001% Pluronic F-68, 200 mM NaCl) and concentration using Amicon Ultra-15 filter units (Millipore Sigma). The purified viruses were titered using a SYBR green (Qiagen) quantitative PCR strategy using PCR primers designed to anneal to the WPRE element present in both viruses: forward primer, CCGTTGTCAGGCAACGTG; and reverse primer, AGCTGACAGGTGGTGGCAAT. The viruses were diluted to a titer of ∼1 × 10^13^ GC/ml.

### ICMS motor mapping

Within 24 h of the final training-paired stimulation session, ICMS was performed as previously described (Tseng et al., 2020, 2021; Brougher et al., 2021). Rats were anesthetized with ketamine hydrochloride (70 mg/kg) and xylazine (5 mg/kg) injected intraperitoneally and placed in a stereotaxic frame. Fiber-optic cannulae were surgically removed. A small incision was made in the cisterna magna to prevent cortical swelling. A craniotomy and durotomy were performed to expose the left motor cortex (ca. +4.0 mm to −4.0 mm AP and +0.2 mm to −5.0 mm ML from bregma). Silicone fluid (Dow Silicones) was used to cover the exposed cortical surface. A tungsten electrode (FHC) with low impedance (0.3–0.8 MΩ, FHC) was lowered 1.8 mm into the left motor cortex, targeting the deep layers of M1, at multiple sites in a grid pattern with 0.5 mm spacing. ICMS consisted of a 40 ms train of ten 200 μs monophasic cathodal pulses delivered at 300 Hz. Stimulation was increased from 0 to 200 μA until a movement was first observed or maximal amplitude was reached. Threshold-evoked movement responses were classified into one of the following categories: proximal FL, distal forelimb (DFL), vibrissa, jaw, neck, trunk, hindlimb, or tail. If no movement was observed at 200 μA, responses were then evaluated at 1.6 mm and 2.0 mm electrode depths. Stimulation sites were tested in random order, and the borders of the motor cortex were defined by unresponsive sites at 200 μA amplitude at all three depths. ICMS was performed with two experimenters. One experimenter determined the random placement of the stimulation electrode. The other experimenter, blinded to both the location of the electrode and the treatment condition of the rat, delivered the stimulation and determined the primary movement and threshold stimulation amplitude.

### Electrophysiology

Three untrained female TH-Cre + rats, aged 33–68 weeks old on the day of recording, received ChR2 virus infusion into the left LC, as described above. At least 4 weeks after the virus infusion, rats were anesthetized with ketamine hydrochloride (70 mg/kg) and xylazine (5 mg/kg) injected intraperitoneally and received supplementary doses of ketamine hydrochloride (70 mg/kg) as needed to maintain stable anesthesia throughout the recording session. A small craniotomy was made to target the LC (AP, −12 mm; ML, −1.25 mm from Bregma), and an optrode consisting of a 200 µm core optical fiber (Thorlabs) glued to a high-impedance (∼1 MΩ) bipolar tungsten matrix microelectrode (FHC; SKU 30255) was slowly lowered to 5.2 mm from the surface of the brain at an angle of 20° posterior to the vertical axis. Neural activity was then recorded every 100–200 μm along the recording track from depths between 5.2 and 6.8 mm. Putative LC neural activity was identified by stereotaxic recording depth, the presence of long-duration positive–negative action potential waveforms, and a burst of spikes following toe pinch (Martins and Froemke, 2015; McCall et al., 2015; Hulsey et al., 2017). During recordings, 0.5 s trains of 473 nm laser pulses were delivered with a random intertrial interval between 10 and 16 s. Different frequencies of stimulation (3, 10, or 30 Hz) were randomly interleaved during recording. Neural activity was recorded using a Plexon OmniPlex Data Acquisition System (Plexon). Wide-band continuous activity was filtered from 0.1 Hz to 10 kHz and digitally sampled at 40 kHz. To capture single-unit activity, wide-band signals were digitally filtered (50 Hz–10 kHz), and spikes were sampled at 40 kHz for a duration of 2.5 ms around the time when a voltage threshold crossing was detected. Following all recording experiments, histology was performed to confirm the anatomical position of the optrode and virus expression in the LC.

Recorded spikes were manually sorted offline into clusters using Plexon Offline Sorter (Plexon). Data analysis was performed in MATLAB. Clusters exhibiting low tonic firing rates (<9 Hz) and long-duration positive–negative action potential waveshapes (peak duration >375 μs) were identified as putative noradrenergic LC units and accepted for further analysis if a unit activity was stably recorded during at least 24 trials. To determine whether each unit exhibited stimulation-evoked responses, peristimulation time histograms were constructed from −1 to +3 s around stimulation train onset, using a 100 ms bin width. Peristimulus time histograms (PSTHs) were constructed for all trial types together, as well as separately for 3, 10, and 30 Hz stimulation trials, and were smoothed using a three-bin moving average. The baseline firing rate for each unit was computed from the smoothed all-trial PSTH during a 0.5 s window prior to stimulation (−0.6 to −0.1 s from train onset). Units were identified as having laser-driven spikes in response to 3, 10, or 30 Hz stimuli if the stimulation-specific PSTH exceeded two standard deviations above the baseline firing rate in any bin during the 0.5 s window corresponding to train presentation (0 to +0.5 s from train onset). Units were identified as exhibiting laser offset-evoked pause responses at each frequency if the stimulation-specific PSTH was two standard deviations below the baseline firing rate for at least 300 ms during the 0.5 s window poststimulation (+0.5 to +1.0 s from the train onset).

We further characterized the stimulation frequency-specific burst-pause responses of identified LC neurons. For each LC unit, the mean number of spikes evoked in the initial laser-evoked burst of firing was computed using the first 0.2 s of train presentation (0 to +0.2 s from the train onset). The mean firing rate during the baseline period (−0.6 to −0.1 s from train onset) was subtracted from the mean firing rate during the 0.2 s burst period to compute the laser-evoked change in firing rate at each frequency. Pause durations were calculated for each unit and stimulation frequency from the stimulation-specific smoothed PSTHs. For each PSTH, pause duration was estimated as the number of consecutive post-train onset bins (× 100 ms) in which the firing rate fell at least two standard deviations below the baseline firing rate or was equal to 0. For each PSTH, the first bin after stimulation onset that met these threshold criteria was used as the start of the pause response included in the analysis.

### Histology

Immediately after the completion of ICMS mapping or electrophysiology recordings, rats were deeply anesthetized with >150 mg/kg sodium pentobarbital and phenytoin solution delivered intraperitoneally and transcardially perfused with 120 ml ice-cold PBS followed by 120 ml 4% paraformaldehyde in PBS. Brains were removed and stored in 4% paraformaldehyde overnight for fixation. The following day, brains were transferred to 30% sucrose for 48–72 h for cryoprotection. A Leica CM1860 cryostat was used to make coronal sections containing the LC (−9.5 to −10.3 mm from Bregma) at 70 μm thickness. Free-floating slices were washed 1× in PBS, followed by permeabilization with 0.5% Triton-X100 in PBS for 30 min. Slices were then washed and blocked in 2.0% BSA in PBS for 1 h. After a series of washes in PBS, slices were incubated overnight at 4°C in a primary antibody cocktail to label tyrosine hydroxylase (TH) and to intensify the eYFP labeling (chicken anti-TH, 1:1,000 dilution, Abcam #ab76442; rabbit anti-GFP, 1:1,000 dilution, MBL #598). The following day, slices were washed 3× in PBS and then incubated at room temperature for 1 h in secondary antibody solution (anti-chicken IgY conjugated to Alexa Fluor 555, 1:2,000 dilution, Abcam #ab150170; anti-rabbit IgG conjugated to Alexa Fluor 488, 1:2,000 dilution, Abcam #ab150081). Finally, slices were washed 3× in PBS and mounted on slides in a DAPI-containing mounting medium (DAPI Fluoromount-G).

For each subject, three different slices of the LC were imaged at 2× or 4× magnification to confirm fiber placement and at 20× to quantify virus expression. All imaging was performed with an Olympus BX51 fluorescent microscope, and image quantification was performed using MATLAB. For each 20× TH and GFP image pair, both images were first converted to 8-bit grayscale, and a region of interest (ROI) was obtained using TH fluorescence as an indicator of LC nucleus boundaries (threshold = 80). The ROI was then applied as a mask to the GFP image of the same slice. The mean gray value (MGV) of the GFP image was computed within the ROI to estimate fluorescence intensity in the LC. Background MGV was similarly calculated for the entire GFP image, excluding the LC ROI and any regions containing the 4th ventricle. The ratio of LC MGV to background MGV was computed for each of the three slices per subject and averaged to quantify LC virus expression in each rat. Subjects were excluded from data analysis if the average signal-to-background MGV ratio did not exceed 1.40 (i.e., included subjects exhibited GFP fluorescence in the LC that was at least 40% higher than the background fluorescence).

### Statistical analysis

Statistical analyses of ICMS mapping data and lever-pressing behavior were performed in MATLAB. All summary data are presented as mean ± standard error of the mean (SEM). The results of ICMS mapping were first compared using a two-way analysis of variance (ANOVA) to analyze the main effects of sex and LC stimulation frequency on motor map plasticity, followed by Bonferroni-corrected *post hoc t* tests. Between-group differences are reported as significant for Bonferroni-corrected *p* < 0.05.

For each rat, the average percent correct performance and average number of trials per session were calculated across the five sessions prior to stimulation (pre-stimulation period) and across the five sessions of training-paired stimulation (stimulation period). The lever-pressing speed for each trial was computed as the maximum lever speed during the 200 ms window following trial initiation. Pressing speeds were averaged across all trials for each session and then across sessions to obtain estimates of pressing speed during the pre-stimulation and stimulation periods. For all behavioral parameters examined, paired *t* tests were used to test for changes in task performance between the pre-stimulation and stimulation periods, and two-way ANOVAs were used to test for specific effects of sex and LC stimulation frequency.

## Results

To selectively stimulate noradrenergic neurons in the LC (LC-NA), we used a viral approach to achieve Cre-dependent, eYFP-tagged channelrhodopsin (ChR2) expression in the LC of TH-Cre rats trained on a lever-pressing task (Fig. 1). Histological analyses confirmed the placement of optical fibers above the LC, and eYFP labeling restricted to TH-positive noradrenergic LC neurons for all rats included in the study (Fig. 1*E*). We also verified that total task exposure (Fig. 1*F*) and subjects’ ages at the time of treatment and mapping (Fig. 1*G*) were balanced across groups. Three female rats included in the study received stimulation at >50 weeks old, leading to a difference in the average ages of male versus female rats at the time of ICMS mapping. However, across treatment groups, neither the total number of training sessions performed nor the subjects’ ages were found to differ significantly, nor were any interaction effects between treatment group and sex observed [total sessions performed: sex effect, 0.394 (*F* = 6.69), treatment effect, *p* = 0.298 (*F* = 1.08), interaction, *p* = 0.461 (*F* = 1.93); age at ICMS: sex effect, *p* = 0.016 (*F* = 8.44), treatment effect: *p* = 0.066 (*F* = 2.73), interaction: *p* = 0.152 (*F* = 1.93)].

### LC-NA stimulation paired with lever pressing drives frequency-dependent M1 map plasticity

**Figure 2.**
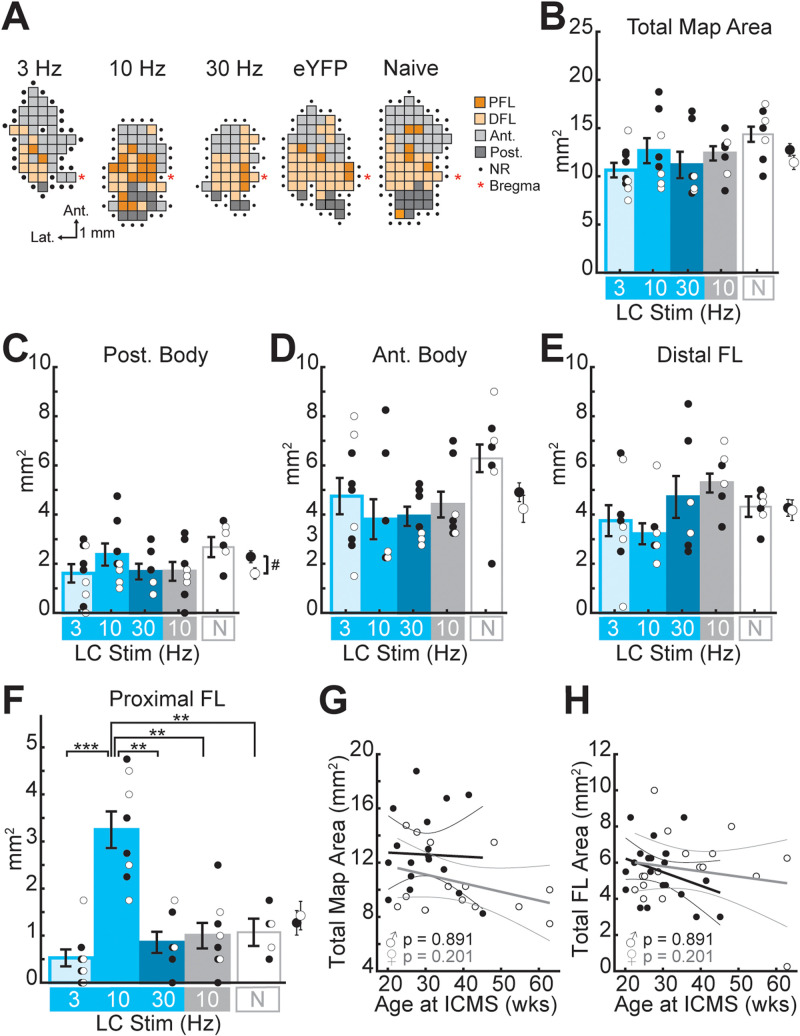
Optogenetic LC-NA stimulation paired with lever pressing induces frequency-dependent plasticity in M1. ***A***, Cortical motor maps from representative subjects in each treatment group that exhibited the median total map area and PFL representation for that group. DFL, distal forelimb; Ant., anterior body (vibrissa + jaw + neck); Post., posterior body (trunk + hindlimb + tail); NR, nonresponsive. Scale for all maps shown below the 3 Hz map. Maps for all animals in the study are shown in Figure 3. ***B***,***C***, Optogenetic LC stimulation does not significantly alter the total motor map area (***B***) or posterior (Post.) body representations (***C***). For all bar plots, cyan bars represent ChR2-expressing rats that received 3, 10, or 30 Hz (as labeled) laser stimulation of the LC, the gray bar represents the eYFP-expressing control group that received 10 Hz laser stimulation, and the white bar on the right represents training- and stimulation-naive (N) group. Error bars denote SEM. Dots represent individual data of male (filled/black dots) and female (open/white dots) rats; averages for all male and female rats are plotted to the right of each bar plot. Male rats were found to have larger posterior body representations (***C***). ^#^*p* < 0.05, sex effect, two-way ANOVA. ***D***,***E***, No significant effects of LC stimulation or sex were seen for anterior (Ant.) body representations in M1 (***D***) or for distal forelimb (FL) representations (***E***). ***F***, Skilled lever pressing paired with phasic 10 Hz optogenetic LC-NA stimulation resulted in a significant enlargement of the task-relevant proximal FL representation in M1 compared to all other treatment groups. **p < 0.01, ***p < 0.001, Bonferroni-corrected *t* tests. ***F***,***G***, Age at ICMS mapping was not significantly correlated with total map size (***G***) or total forelimb (FL) map area (***F***).

**Figure 3.**
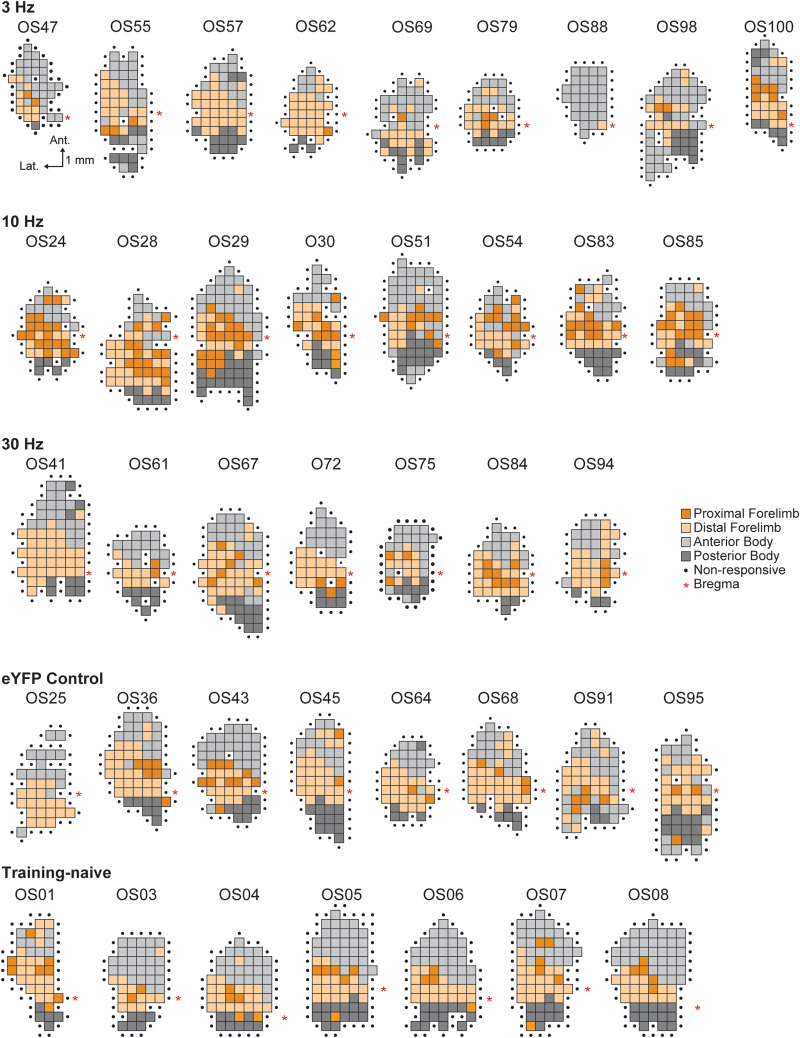
Cortical motor maps from all subjects. For animals that underwent lever training and training-paired LC-NA stimulation, motor maps were obtained via ICMS within 24 h of the last LC stimulation paired training session. The scale bar for all maps is shown under the map at the top left (OS47); the legend for all maps is shown to the right of the 30 Hz group (middle row).

To determine whether task-paired LC-NA stimulation is sufficient to induce motor cortical map reorganization, ChR2-expressing TH-Cre rats received optogenetic LC-NA stimulation at 3 Hz (*n* = 9), 10 Hz (*n* = 8), or 30 Hz (*n* = 7) paired with correct motor performance of a well-learned skilled reaching lever-press task. A control group of eYFP-expressing TH-Cre rats (*n* = 9) received training-paired 10 Hz stimulation. Rats received five training sessions in which optogenetic LC-NA stimulation was paired with each correct lever press. Somatotopic motor maps were obtained within 24 h of the last training-paired stimulation session (Figs. 2*A*, 3). As cortical motor maps are known to exhibit transient task-dependent plasticity during initial task learning (Conner et al., 2003; Monfils et al., 2005; Molina-Luna et al., 2008; Tennant et al., 2012), maps were obtained from an additional group of training-naive, unstimulated rats (*n* = 7), to ensure that maps in our extensively trained animals did not differ from those of untrained subjects. Motor maps were compared across treatment groups to examine the frequency-dependent effects of LC-NA stimulation on M1 map plasticity (Fig. 2*B–F*).

We performed two-way ANOVAs to test for the effects of sex and stimulation treatment on motor map organization (Table 1). Consistent with our previously published findings (Tseng et al., 2020), posterior body representations (trunk + hindlimb + tail) were found to be significantly larger among males compared to females (Fig. 2*C*, Table 1). No other subregions were found to exhibit significant sex-specific differences in map representation (Fig. 2*D–F*, Table 1) nor were any interaction effects observed between sex and LC-NA stimulation (Table 1).

**Table 1.** Results of two-way ANOVA testing for effects of sex and stimulation treatment on cortical motor map representations

	Two-way ANOVA		
	*p*_sex_ [*F*_sex_]	*p*_stim_ [*F*_stim_]	*p*_interaction_ [*F*_interaction_]
Total map area (mm^2^)	0.172 [1.96]	0.074 [2.39]	0.186 [1.66]
Posterior body area (mm^2^)	**0.038 [4.72]**	0.089 [2.24]	0.167 [1.74]
Anterior body area (mm^2^)	0.300 [1.11]	0.074 [2.39]	0.175 [1.71]
DFL area (mm^2^)	0.956 [0.0]	0.079 [2.34]	0.511 [0.84]
PFL area (mm^2^)	0.891 [0.02]	**0.000 [15.52]**	0.938 [0.2]

ICMS-evoked movements at threshold amplitudes were classified as posterior body (trunk, hindlimb, or tail), anterior body (vibrissa, jaw, or neck), distal forelimb (DFL), or proximal forelimb (PFL) movements. Consistent with prior reports, sex-specific effects were found in the posterior body map area. Stimulation-specific effects were found only for the task-relevant PFL representation. Bold denotes statistical significance (*p* < 0.05).

Across treatment groups, a significant main effect of LC-NA stimulation frequency was observed only for the task-relevant proximal forelimb (PFL) representation (Fig. 2*B–F*, Table 1). *Post hoc* analyses revealed that successful trial performance paired with phasic 10 Hz LC-NA stimulation resulted in the significant enlargement of the PFL representation in rat M1 compared to the eYFP control group (10 Hz vs eYFP: *T* = −4.74, *p* = 0.0043, Bonferroni-corrected *t* test), the training-naive animals (10 Hz vs untrained: *T* = −5.17, *p* = 0.0053, Bonferroni-corrected *t* test), and the ChR2-infused rats that received lower-frequency 3 Hz LC-NA stimulation (10 Hz vs 3 Hz: *T* = −6.35, *p* = 0.0009, Bonferroni-corrected *t* test) or higher-frequency 30 Hz LC-NA stimulation (10 Hz vs 30 Hz: *T* = −5.32, *p* = 0.0024, Bonferroni-corrected *t* test). Stimulation-driven expansion of the task-relevant PFL map representation was only observed for 10 Hz LC-NA stimulation; PFL representations following 3 Hz and 30 Hz stimulation did not differ significantly from eYFP controls (3 Hz vs eYFP, *T* = −1.45, *p* = 1.0; 30 Hz vs eYFP, *T* = −0.41 *p* = 1.0; 3 Hz vs 30 Hz, *T *= −1.14, *p* = 1.0, Bonferroni-corrected *t* tests) or from untrained rats (3 Hz vs untrained, *T *= 2.26, *p* = 0.405; 30 Hz vs untrained, *T *= 0.78, *p* = 1.0, Bonferroni-corrected *t* tests). No significant effects of training-paired LC-NA stimulation were observed for non–task-relevant motor map representations, including DFL, anterior body (vibrissa + jaw + neck), and posterior body representations (Table 1). Because animals spanned a range of ages up to ca. 15 months old at the time of mapping, we tested whether age-related changes in motor map structure could be impacting our results. We found no correlation between age and total map area (Fig. 2*G*) or FL representation size (Fig. 2*H*) for male or female rats, suggesting that age did not significantly impact relevant map structures in our cohort. Nor did excluding the three rats with statistically outlying ages alter the main finding that expansion of the task-relevant proximal FL map representation was induced only in the 10 Hz LC-NA stimulation group.

Combined, our results suggest that noradrenergic LC stimulation paired with performance of a well-learned motor task is sufficient to induce task-specific neuroplasticity in M1. Importantly, LC-NA-driven M1 plasticity is highly dependent on the frequency of LC stimulation, as only phasic-like 10 Hz stimulation was effective at enhancing task-relevant PFL representations, but not lower (3 Hz) or higher (30 Hz) frequency stimulation.

### LC-NA stimulation did not significantly alter task performance

**Figure 4.**
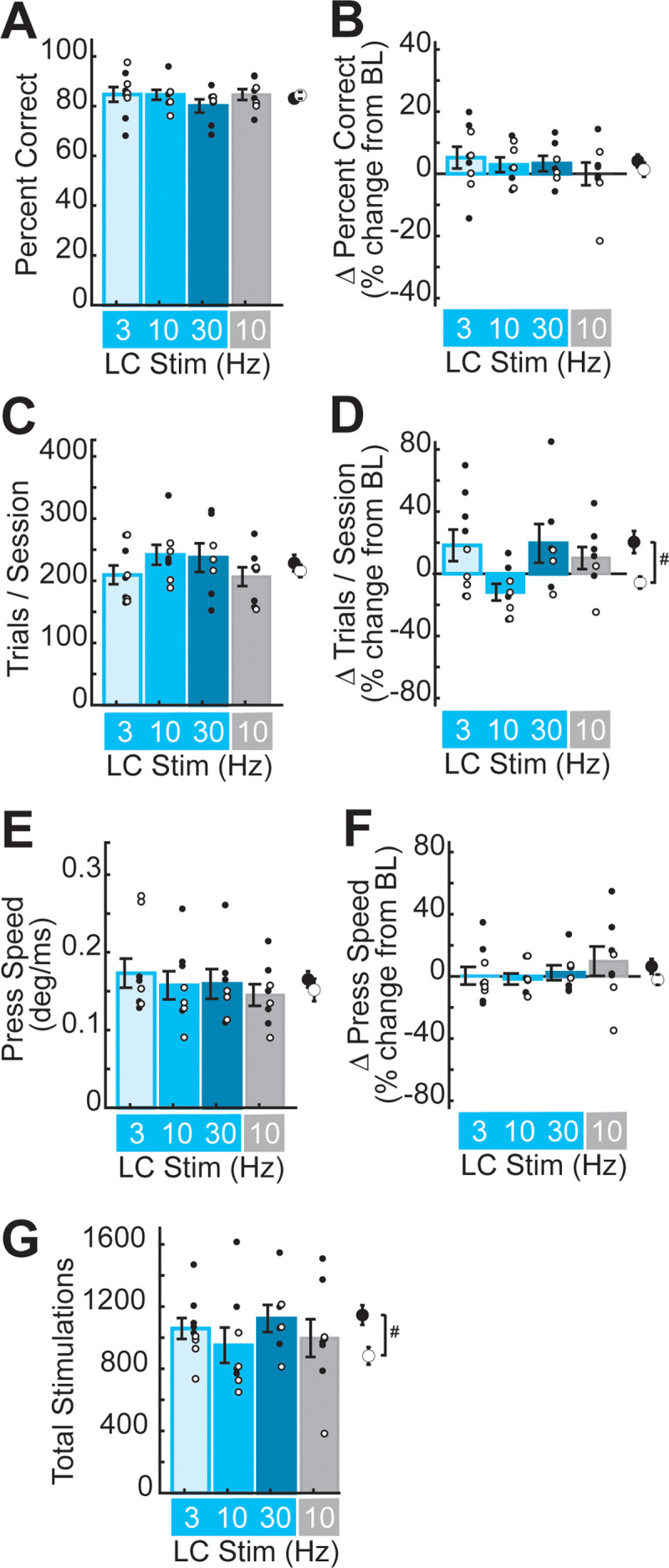
Optogenetic LC-NA stimulation did not significantly impact behavioral performance on the well-learned lever task. ***A***,***B***, Percent correct performance did not differ across sexes or LC-NA stimulation groups during the pre-stimulation period (***A***), nor was percent correct performance altered by LC-NA stimulation (***B***). ***C***, The number of trials performed per session did not differ across sex or LC-NA stimulation groups during the pre-stimulation period. ***D***, During stimulation, we observed a sex-specific difference in task engagement, but no significant differences across LC-NA treatment groups. ^#^*p* < 0.05, sex effect, two-way ANOVA. ***E***,***F***, Lever-pressing speed did not differ across sex or LC-NA stimulation group during the pre-treatment period (***E***) nor did LC-NA stimulation impact pressing speed (***F***). ***G***, The total number of laser stimulation trains delivered did not differ significantly across LC-NA treatment groups but was higher overall in male rats compared to female rats.

To determine whether the frequency-dependent effects of LC-NA stimulation on M1 map plasticity were associated with stimulation-driven changes in behavioral performance, we used two-way ANOVA to test for sex and stimulation-specific effects on behavior in the pre-stimulation and stimulation periods (Table 2). In the five sessions prior to LC stimulation, we found no significant effects of sex or stimulation group on the percentage of correct trials performed (Fig. 4*A*), the total number of lever presses performed per session (Fig. 4*C*), or lever-pressing speed (Fig. 4*E*). Nor was there any interaction between sex and stimulation group during this pre-stimulation training period (Table 2).

**Table 2.** Results of two-way ANOVA testing for effects of sex and LC stimulation frequency on behavior

	Two-way ANOVA		
	*p*_sex_ [*F*_sex_]	*p*_stim_ [*F*_stim_]	*p*_int_ [*F*_int_]
Pre-stimulation baseline performance			
Percent correct	0.668 [0.19]	0.571 [0.68]	0.312 [1.26]
Trials per session	0.436 [0.63]	0.383 [1.06]	0.931 [0.15]
Pressing speed	0.288 [1.18]	0.498 [0.82]	0.056 [2.90]
Performance during stimulation			
Percent correct	0.682 [0.17]	0.234 [1.52]	0.115 [2.20]
Trials per session	**0.008 [8.21]**	0.360 [1.12]	0.960 [0.10]
Total stimulations	**0.011 [7.58]**	0.599 [0.64]	0.941 [0.13]
Pressing speed	0.131 [2.45]	0.789 [0.35]	0.106 [2.27]
Percent change from baseline to treatment period			
Percent correct	0.341 [0.94]	0.585 [0.66]	0.685 [0.50]
NumTrials	**0.007 [8.79]**	0.068 [2.71]	0.710 [0.46]
Pressing speed	0.214 [1.63]	0.838 [0.28]	0.243 [1.49]

During the stimulation period, but not during the pre-stimulation period, a sex-specific effect was observed in the total number of trials performed per session and in the total number of stimulations received. No effects of LC-NA stimulation frequency or sex × stimulation interaction effects were observed for any performance parameter during the pre-stimulation or stimulation periods. Bold denotes statistical significance (*p* < 0.05).

To examine the effects of LC-NA stimulation on behavioral performance, we expressed each rat's performance during the five sessions of training-paired stimulation as a percent change from their performance during the pre-stimulation period. Across all rats, percent correct performance did not change significantly between the pre-stimulation and stimulation periods (percent correct during the pre-stimulation period, 83.6 ± 1.2%; during the stimulation period, 85.8 ± 1.3%, mean ± SEM; *T *= −1.76, *p* = 0.089, paired *t* test). Further, two-way ANOVA showed no significant effects of sex or LC-NA stimulation on percent correct performance (Fig. 4*B*, Table 2), suggesting that during the 5 d treatment period, overall performance accuracy was not significantly impacted by LC-NA modulation at any stimulation frequency.

Across all rats, we found no significant change in the number of trials performed per session between the pre-stimulation and stimulation periods (trials/session during pre-stimulation, 222.8 ± 8.6; during stimulation, 238.9 ± 11.3, mean ± SEM; *T *= −1.72, *p* = 0.095, paired *t* test). However, two-way ANOVA revealed a significant effect of sex on the number of trials performed per session during stimulation, but no significant effect of LC-NA stimulation frequency, and no interaction between sex and stimulation (Fig. 4*D*; Table 2). *Post hoc* analyses confirmed that male rats increased their task engagement during the stimulation period more than female rats (percent change from pre-stimulation in the number of trials per session: male, 20.4 ± 7.10%; female, −5.69 ± 3.83%, mean ± SEM; *T* = −3.23, *p* = 0.0034, Student's *t* test). Male rats typically perform more trials than female rats in this task (Tseng et al., 2020), though it is unclear why this was observed during the stimulation period, but not the pre-stimulation period, in the current study. Regardless, this sex-dependent difference in task engagement was consistent across ChR2 and control groups, suggesting that the effect is unrelated to LC-NA modulation. Consistent with the increase in overall task engagement, male rats on average received a greater number of laser stimulations than female rats (Fig. 4*G*, Table 2). However, the number of stimulations delivered was not correlated with cortical map area for any subregion (number of stimulations vs total map size: *r* = −0.043, *p* = 0.814; posterior body representation, *r* = 0.119, *p* = 0.518; anterior body representation, *r* = 0.025, *p* = 0.894; DFL representation, *r* = −0.020, *p* = 0.913; PFL representation, *r* = −0.200; *p* = 0.271; Pearson's correlation), indicating that the frequency-specific LC-NA-driven M1 plasticity we observed does not depend on the specific number of movement-paired stimulations received during training.

We next tested the impact of LC-NA stimulation on lever-pressing speed. Across all rats, we found no significant change in pressing speed between the pre-stimulation and stimulation periods (press speed during pre-stimulation, 0.159 ± 0.009 deg/ms; during stimulation, 0.164 ± 0.010 deg/ms, mean ± SEM; *T *= −0.904, *p* = 0.373, paired *t* test). Nor did we find a significant effect of sex or stimulation frequency on pressing speed during stimulation (Fig. 4*F*, Table 2), indicating that LC-NA activation did not alter gross motor coordination or movement speeds.

Taken together, our results indicate that training-paired LC-NA stimulation was not associated with significant changes in lever-pressing performance at any frequency. Nor can changes in behavioral performance explain the frequency-dependent neuroplastic effects of LC stimulation that we observed in M1. These behavioral findings suggest that driving phasic LC-NA signaling during experience-dependent M1 activation is sufficient to induce reorganization of cortical motor maps, even late in training after neural representations and behavioral performance are typically optimized and stable (Li et al., 2001; Xu et al., 2009; Huber et al., 2012; Peters et al., 2014).

### Optogenetic stimulation drives frequency-dependent LC-NA firing dynamics

**Figure 5.**
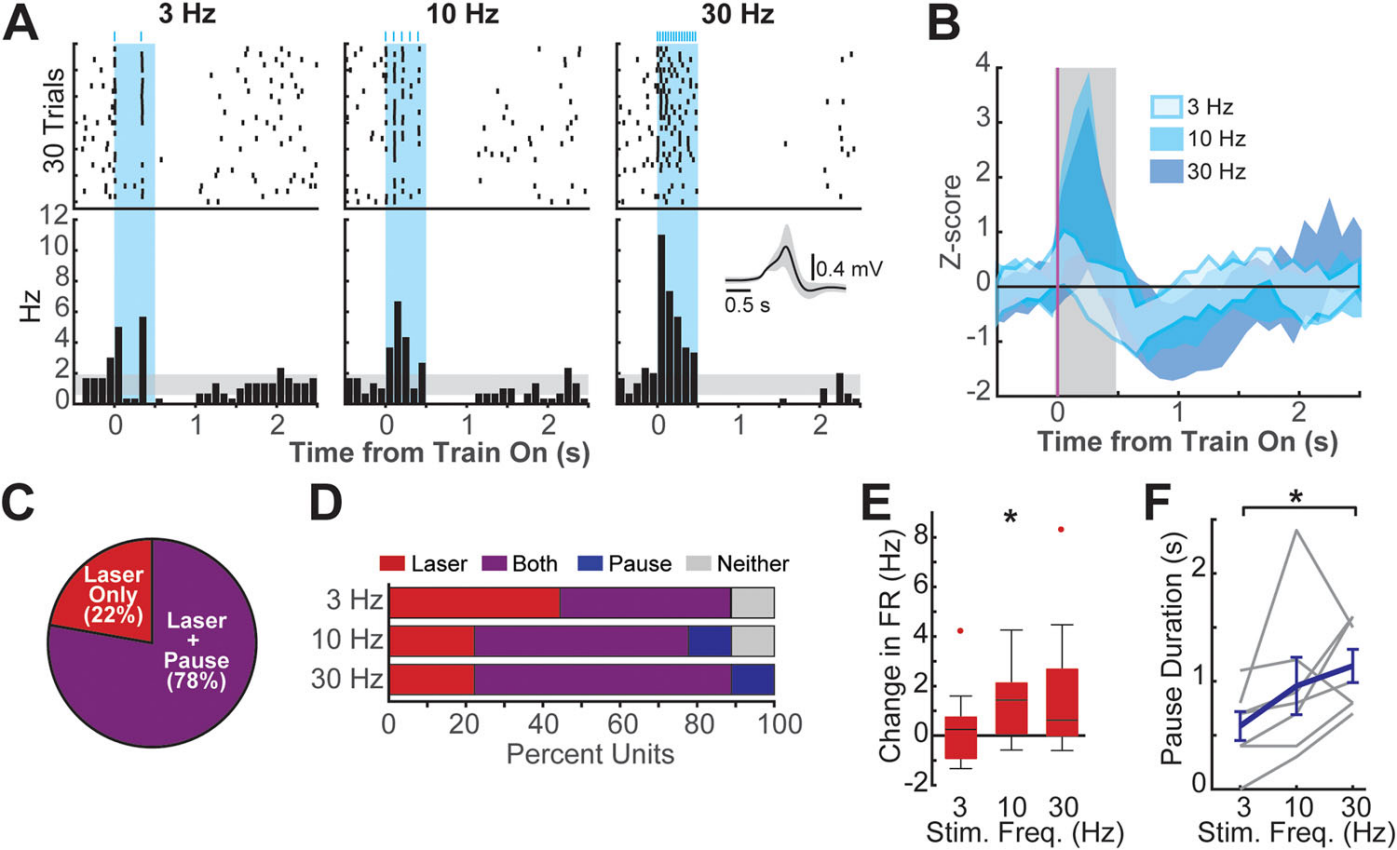
Optogenetic stimulation drives LC-NA burst-pause activity. ***A***, Peristimulation time histogram and raster plots of an example LC-NA neuron that exhibited both laser-evoked firing and poststimulation pauses. ***B***, Average *z*-score normalized responses to 3, 10, and 30 Hz stimulation across all nine recorded LC-NA units. The shaded area represents mean population activity ± SEM. ***C***, All LC units exhibited laser-evoked increases in firing during the 0.5 s laser pulse train, and the majority additionally exhibited a pause in firing following train offset. ***D***, More units exhibited pause responses as the stimulation frequency increased from 3 to 30 Hz. ***E***, Across the population of LC units, laser stimulation at 10 Hz, but not at 3 or 30 Hz, significantly increased firing rates during the initial 200 ms after train onset. *p < 0.05, one-sample *t* test. ***F***, Increasing stimulation frequency resulted in an increase in pause durations. *p < 0.05, Bonferroni-corrected *t* tests.

To characterize the single-unit responses of LC-NA neurons to the delivery of 0.5 s trains of 3, 10, and 30 Hz optogenetic stimulation, we performed in vivo electrophysiological recordings in anesthetized rats (Fig. 5*A*). The stimulation frequency-dependent firing of nine putative LC-NA neurons was recorded at seven recording sites in three rats. Across the population, significant laser-driven spiking during train delivery was observed, as well as a significant poststimulation pause in firing (Fig. 5*B*). Optogenetic stimulation drove laser-evoked spiking in all LC-NA neurons recorded (9/9%, 100%), and the majority of neurons also exhibited significant poststimulation pause responses (7/9%, 77.8%; Fig. 5*C*). The number of LC-NA neurons exhibiting laser-evoked responses was similar across 3, 10, and 30 Hz stimulation frequencies; however, more neurons exhibited significant poststimulation pause responses as stimulation frequency increased (Fig. 5*D*).

We next tested whether the intensity of laser-evoked spiking or pause responses varied with stimulation frequency. LC-NA firing rates were seen to accommodate rapidly following a peak around ca. 200 ms after laser onset (Fig. 5*A*,*B*). As this rapid accommodation may be the result of both the physiological properties of LC-NA neurons (Berridge and Waterhouse, 2003) as well as limitations of the hChR2(H134R) opsin (Berndt et al., 2011; Lin, 2011), we focused on the initial 200 ms following train onset to test whether the laser-evoked burst of LC-NA firing exhibited frequency dependence. We found that only the 10 Hz phasic-like stimulation drove a burst of firing across the population that was significantly greater than the baseline firing rate (Fig. 5*E*; laser-evoked change in firing rate at 10 Hz: 1.37 ± 0.58 spikes/s, mean ± SEM; *T *= 2.37, *p* = 0.045, one-sample *t* test). By contrast, tonic-like 3 Hz stimulation did not significantly impact LC-NA firing rates (change in firing rate at 3 Hz: 0.44 ± 0.56 spikes/s, mean ± SEM; *T *= 0.79, *p* = 0.454, one-sample *t* test), whereas responses to 30 Hz “overstimulation” were highly variable, resulting in no net increase in firing across the population of recorded LC-NA cells (change in firing rate at 30 Hz: 1.77 ± 0.97 spikes/s, mean ± SEM; *T *= 1.83, *p* = 0.104, one-sample *t* test). Among the LC-NA neurons that exhibited pause responses, poststimulation pause durations were found to significantly increase as stimulation frequency increased (Fig. 5*F*; pause durations: 3 Hz, 0.56 ± 0.13 s; 10 Hz, 0.96 ± 0.27 s; 30 Hz, 1.14 ± 0.15 s, mean ± SEM; 3 vs 10 Hz, *T* = −1.77, *p* = 0.2517; 3 vs 30 Hz, *T* = −3.05, *p* = 0.0448, Bonferroni-corrected paired *t* tests).

Our recording results demonstrate that the optogenetic stimulation paradigm used in this study transiently activates burst-pause responses in LC-NA neurons. Moreover, our results suggest that increasing intensities of optogenetic LC-NA stimulation are likely to produce brief increases in evoked firing at frequencies that are consistent with a physiologically plausible phasic firing mode. Further increases in stimulation intensity then shift LC-NA responses toward a stimulation-driven silencing of the LC-NA population. These stimulation frequency-specific LC-NA firing dynamics likely contribute to the inverted U-shaped ability of LC stimulation to induce motor map plasticity as stimulation frequency is increased.

## Discussion

Our findings demonstrate that LC-NA stimulation is sufficient to induce task-relevant plasticity in the motor cortex. Moreover, we find that this LC-NA stimulation-induced plasticity exhibits an inverted U-shaped relationship with stimulation frequency. Prior studies have shown that LC-NA stimulation paired with training can enhance sensory representations in granular sensory cortices and in the hippocampus (Manunta and Edeline, 2004; Devilbiss and Waterhouse, 2011; Martins and Froemke, 2015; Mizuyama et al., 2016; Wagatsuma et al., 2017; Gelbard-Sagiv et al., 2018; Glennon et al., 2019; Grella et al., 2019; Kaufman et al., 2020; McBurney-Lin et al., 2022). Further, now-classic studies demonstrate that prefrontal cortex-dependent attentional performance exhibits a well-known inverted U-shaped relationship to LC firing frequency (Usher et al., 1999; Levy, 2009; Devilbiss, 2019). Our current findings provide a novel extension of this previous work by demonstrating the frequency dependence of LC-NA stimulation-induced map plasticity in agranular M1, with implications for understanding the neural circuits impacting motor learning in health and disease.

### Learning is associated with cortical map reorganization

Reorganization of cortical maps has been shown to be associated with perceptual learning and motor skill learning (Jenkins et al., 1990; Conner et al., 2003; Bao et al., 2004; Doyon and Benali, 2005; Monfils et al., 2005; Molina-Luna et al., 2008; Pienkowski and Eggermont, 2011; Reed et al., 2011; Tennant et al., 2012; Harding-Forrester and Feldman, 2018). Expansion of task-relevant map representations generally accompanies improvement of sensory or motor task performance during initial learning stages, and blocking cortical map reorganization has been shown to result in failure to acquire a new task (Conner et al., 2003, 2010). Once subjects master a behavior, however, cortical maps revert to a structure that is similar to that of task-naive subjects, while skilled performance remains stable (Molina-Luna et al., 2008; Reed et al., 2011; Porter et al., 2012; Tennant et al., 2012). These findings suggest that the reorganization of the cortical maps reflects neural plasticity that is necessary for acquiring a new task but not for maintaining behavioral proficiency once the subject has learned.

In the current study, we find that phasic LC-NA stimulation paired with a well-learned motor task-induced task-relevant plasticity in M1 without significantly altering behavioral performance. While this finding is consistent with the studies summarized above, which demonstrate that expert performance late in training is stably maintained independent of cortical map structure, other studies have reported improved behavioral performance following LC stimulation or administration of pharmacological agents that manipulate noradrenergic signaling (Martins and Froemke, 2015; Mizuyama et al., 2016; Navarra et al., 2017; Wagatsuma et al., 2017; Gelbard-Sagiv et al., 2018; Glennon et al., 2019). In these studies, improved behavioral performance can be attributed to an enhanced ability to detect or discriminate sensory or contextual cues or to accelerated updating of neural representations following rule-switching. Enhanced sensory discrimination and behavioral flexibility are thought to arise as a result of NA-mediated gating of cortical excitability, which sharpens sensory tuning, improves signal-to-noise ratios, and promotes task-relevant synaptic plasticity (Berridge and Waterhouse, 2003; Salgado et al., 2016; Jacob and Nienborg, 2018; McBurney-Lin et al., 2019). Our results point to the existence of similar NA-mediated gating of cortical activity in M1. Here, 24 h after the last task-paired LC-NA stimulation session, we still observe significant motor map reorganization in the 10 Hz stimulation group, suggesting that phasic NA release during training enhances ongoing task-relevant M1 activity and promotes long-term cortical plasticity. The absence of plasticity-related behavioral effects in the current study may additionally arise in part due to the nature of our training paradigm, which did not impose stringent demands on movement speeds, intertrial intervals, or attention, for example. As a result, behavioral variability is high even among the well-trained rats in our study, and this could have masked more subtle effects of enhanced neuromodulation in M1. Going forward, it will be important to fully test the functional implications of NA-driven alterations in M1 processing and plasticity using more sensitive motor tasks or learning paradigms specifically designed to address this issue.

### Motor cortical plasticity enhances functional recovery after a stroke

Neural injuries such as stroke are often associated with deficits in motor function and with cortical reorganization within the intact and peri-lesion motor cortical areas (Dimyan and Cohen, 2011; Hosp and Luft, 2011; Caleo, 2015; Okabe et al., 2016). After a stroke, spontaneous recovery of cortical representations of the impaired musculature can be observed but is typically limited. Motor rehabilitation consisting of enforced usage of the affected musculature has been shown to enhance cortical reorganization and restoration of motor function after injuries (Liepert et al., 2000; Caleo, 2015; Okabe et al., 2016). Rehabilitation-induced motor functional recovery is disrupted when cortical reorganization is inhibited (Conner et al., 2005; Okabe et al., 2016; Meyers et al., 2019), suggesting the importance of neuroplasticity as a substrate for rehabilitation-induced functional recovery. Therapies that can enhance experience-dependent neuroplasticity, including transcranial magnetic stimulation, VNS, and deep brain stimulation have been explored to further improve the efficacy of post-stroke motor rehabilitation (Elias et al., 2018; Schambra, 2018; Engineer et al., 2019; Ramos-Castaneda et al., 2022; Starosta et al., 2022; Ananda et al., 2023).

The current study follows prior work from our lab and others demonstrating that training-paired VNS induces NA-dependent motor cortical plasticity (Hulsey et al., 2019; Tseng et al., 2021), which is associated with enhanced functional recovery following stroke (Khodaparast et al., 2013; Meyers et al., 2018; Dawson et al., 2021; Pruitt et al., 2021). In preclinical rodent models, VNS-driven neuroplasticity and enhanced stroke recovery exhibit identical inverted U-shaped dose dependence (Morrison et al., 2019; Pruitt et al., 2021). In this context, our current results indicate that phasic 10 Hz LC activation is sufficient to reproduce the neuroplastic effects of VNS, reinforcing the critical contribution of noradrenergic signaling to VNS efficacy. Our findings, moreover, predict that rehabilitation-paired 10 Hz LC-NA stimulation should induce similar long-lasting improvements in motor function during stroke recovery. Although optogenetic techniques enable precise temporal control of targeted neurons in preclinical research (Tye and Deisseroth, 2012), the use of optogenetics in clinical applications remains limited due to safety concerns and technical challenges (Shen et al., 2020). While progress is being made to overcome these challenges, pharmacological manipulations of noradrenergic signaling, alone or in combination with a temporally precise therapy such as VNS, may provide a more approachable means to enhance therapeutic neuroplasticity during injury recovery.

### Dose-dependent effects of noradrenergic activity

In nonmotor cortical regions, NA has long been known to exhibit inverted U-shaped dose-dependent effects on neuronal excitability, synaptic plasticity, perceptual learning, and attention (Aston-Jones and Cohen, 2005; Levy, 2009; Devilbiss, 2019). In the current study, we find a similar dose-response relationship between LC-NA activation and cortical plasticity in the agranular motor cortex. Here, 10 Hz phasic LC-NA stimulation was sufficient to drive task-specific motor cortical map reorganization, whereas 3 and 30 Hz LC-NA stimulation failed to induce M1 plasticity. When combined with published literature, these results point to the inverted U-shaped Yerkes–Dodson relationship as a general principle governing not just short-term NA-gated cortical arousal or attention but long-term NA-mediated experience-dependent optimization of cortical circuit function across structural variation and functional domains.

Though the precise mechanisms underlying LC-NA-driven cortical plasticity may vary across cortical regions, developmental stages, and/or behavioral contexts, there are several local cellular and synaptic processes within the neocortex that could contribute to the U-shaped dose-dependent effects that we observed. Low versus high concentrations of NA are known to differentially engage high-affinity α versus low-affinity β adrenergic receptors (Molinoff, 1984), which are widely expressed on cortical projection neuron populations and generally exert opposing effects on neuronal excitability and synaptic plasticity (Salgado et al., 2016). Cortical interneuron populations also differentially express α and β adrenergic receptors (Kawaguchi and Shindou, 1998; Liu et al., 2014; Tasic et al., 2016), and feedforward and feedback inhibition may thus be dose-dependently engaged. At high cortical NA concentrations, overstimulation of G-protein coupled adrenergic receptors may result in desensitization (Racagni et al., 1983; Sulser et al., 1984), contributing to an inverted U-shaped dose-dependent circuit performance.

NA is known to provide strong neuromodulation of M1 circuits, and we have previously shown that cortical noradrenergic signaling is required for a very similar type of stimulation-driven map plasticity induced by VNS (Hulsey et al., 2019; Tseng et al., 2021). These prior findings suggest that frequency-dependent modulation of M1 noradrenergic signaling plays a key role in generating the task-specific, stimulation frequency-dependent motor map plasticity that we observed. However, efferent fibers from the LC are known to project to nearly all areas in the CNS (Aston-Jones and Waterhouse, 2016; Poe et al., 2020), and we cannot rule out the possibility that indirect pathways also contribute to our findings. Multiple M1 input regions also undergo NA-dependent neuromodulation, including the cerebellum, thalamus, and prefrontal cortex (Berridge and Waterhouse, 2003; Waterhouse et al., 2022), and facilitation of signal transmission throughout the motor network could contribute to the enhancement of task-relevant movement representations within M1. The LC also innervates numerous other cortically projecting neuromodulatory nuclei known to impact cortical function and map structures, including the serotonergic raphe nuclei, dopaminergic ventral tegmental area, and the cholinergic basal forebrain (Jones and Moore, 1977; Conner et al., 2010; Hulsey et al., 2019). Further studies are needed to fully elucidate the cellular, synaptic, and network mechanisms underlying LC-NA-driven motor cortical plasticity.

The precise temporal dynamics of LC-NA responses themselves are also known to play a role in determining where along the U-shaped curve a given cortical network is operating. Phasic and tonic firing modes of the LC are known to have distinct modulatory effects on target neuronal circuits (Aston-Jones and Cohen, 2005; Devilbiss and Waterhouse, 2011; Howells et al., 2012; Devilbiss, 2019; Grella et al., 2019). In addition, phasic but not tonic LC activity has been shown to generate neuroplasticity in sensory cortex and in the hippocampus (Manunta and Edeline, 2004; Devilbiss and Waterhouse, 2011; Edeline et al., 2011; Martins and Froemke, 2015; Vazey et al., 2018; Glennon et al., 2019; Grella et al., 2019; Kaufman et al., 2020). In these regions, phasic LC signaling is thought to be linked to optimal task performance by enhancing neural encoding of task-relevant stimuli (Salgado et al., 2016; Jacob and Nienborg, 2018; McBurney-Lin et al., 2019). In the current study, we extend this prior work, finding that brief LC-NA stimulation induces plasticity in M1 cortical representations only when applied at an intermediate frequency that is consistent with physiological phasic LC firing rates.

Our recording results suggest that these frequency-dependent effects may also arise, at least in part, as a result of nonlinear stimulation-induced LC-NA firing dynamics. As stimulation frequency increased, we observed only a modest increase in evoked LC-NA burst firing in response to 10 Hz LC-NA stimulation. As the frequency further increased to 30 Hz, a poststimulation suppression predominated the LC-NA population response. Intrinsic properties of LC-NA cells and/or inhibitory feedback networks may thus result in a brief plasticity-promoting increase in NA release at low-to-moderate levels of phasic stimulation, but a suppression of LC-NA signaling with overstimulation. Such firing dynamics are consistent with known autoinhibitory regulation of LC firing (Egan et al., 1983; Andrade and Aghajanian, 1984; Ennis and Aston-Jones, 1986), for example, though additional research is needed to determine whether natural stimuli evoke similar nonlinearity in the LC-NA burst-pause responses as stimulus intensities increase. We also note that we did not specifically target M1-projecting LC-NA neurons in our recordings, and the extent to which these stimulation-evoked responses vary across different LC-NA projection populations remains to be determined, as does the relevance of such compartmentalization for cortex-dependent behaviors. Understanding the nonlinear and projection-specific dynamics of LC-NA firing will be especially important for the optimization of plasticity-promoting stimulation therapies, such as VNS.

In the current study, we demonstrated that pairing motor experience with phasic LC-NA activation was sufficient to enhance the representation of task-relevant musculature in the agranular motor cortex. Moreover, LC-NA-driven motor cortical plasticity exhibited an inverted U-shaped relationship with increasing stimulation frequency. These findings highlight the temporal dynamics of noradrenergic signaling as an important driver of cortical network optimization and experience-dependent plasticity. Taken together with prior literature, our results suggest that neuromodulatory therapies that enhance phasic cortical noradrenergic signaling during rehabilitation exercises may provide promising treatment strategies for the recovery of motor function after neural injuries such as stroke.
